# Supplementary material for: Evaluation of Orbital Lymphoproliferative and Inflammatory Disorders by Gene Expression Analysis
Source: Int J Mol Sci. 2022 Aug 3;23(15):8609. doi: 10.3390/ijms23158609 (PMC9369106; doi:10.3390/ijms23158609)
Supplement: Supplementary file 1 [file ijms-23-08609-s001.zip › Figures S1, S2 and S3.pdf]

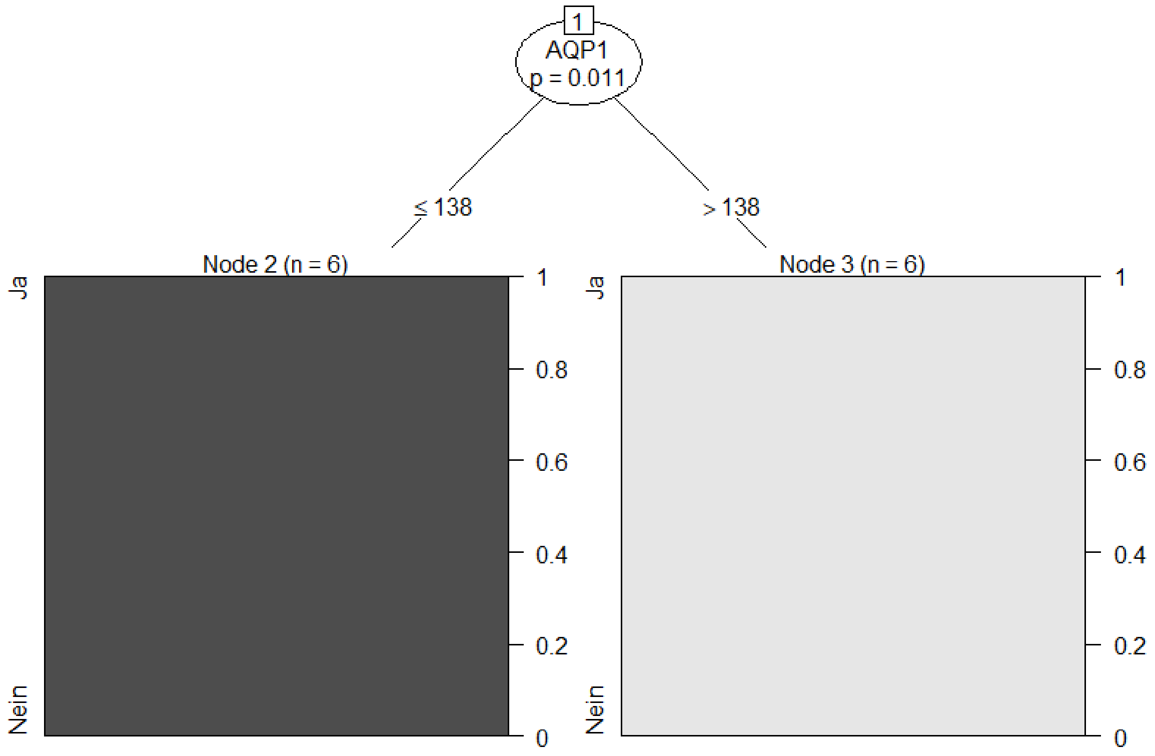

Figure S1. For NSOI, CIT revealed a one-tier system based on *AQP1* expression, with the appertaining cut-off of >138 counts identifying NSOI cases.

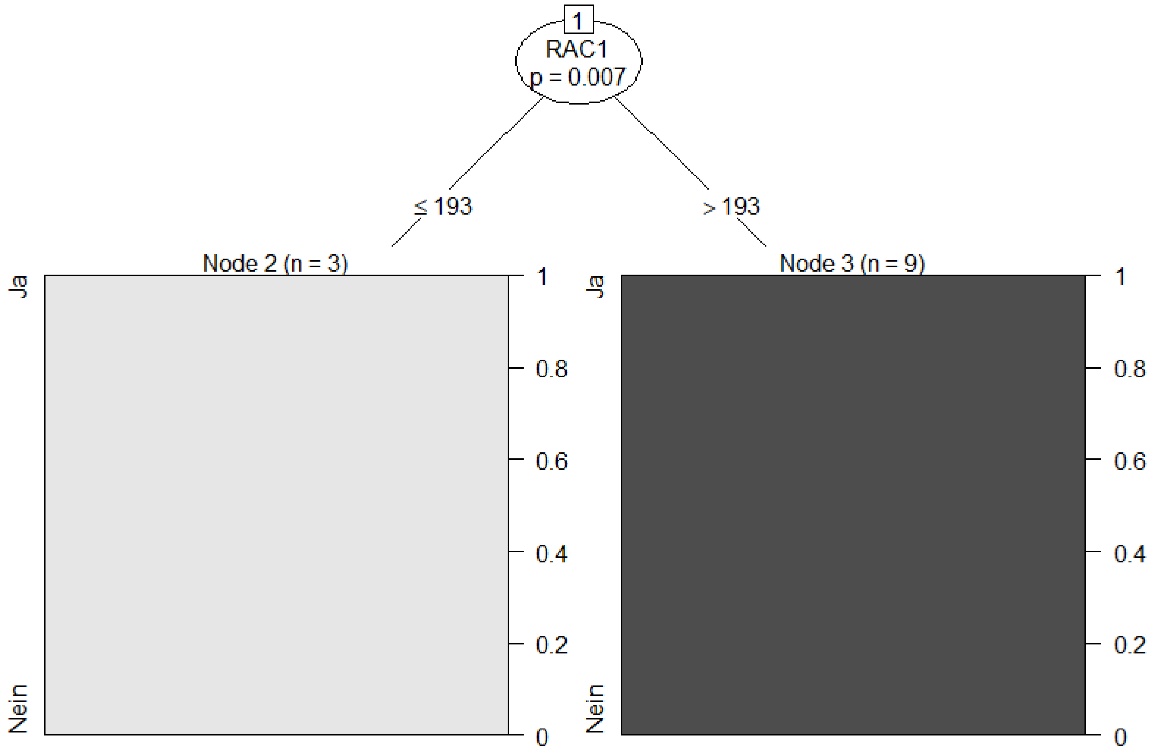

Figure S2. For lymphoma, CIT again revealed a one-tier system based on *RAC1* expression, with the appertaining cut-off of ≤193 counts identifying lymphoma cases.

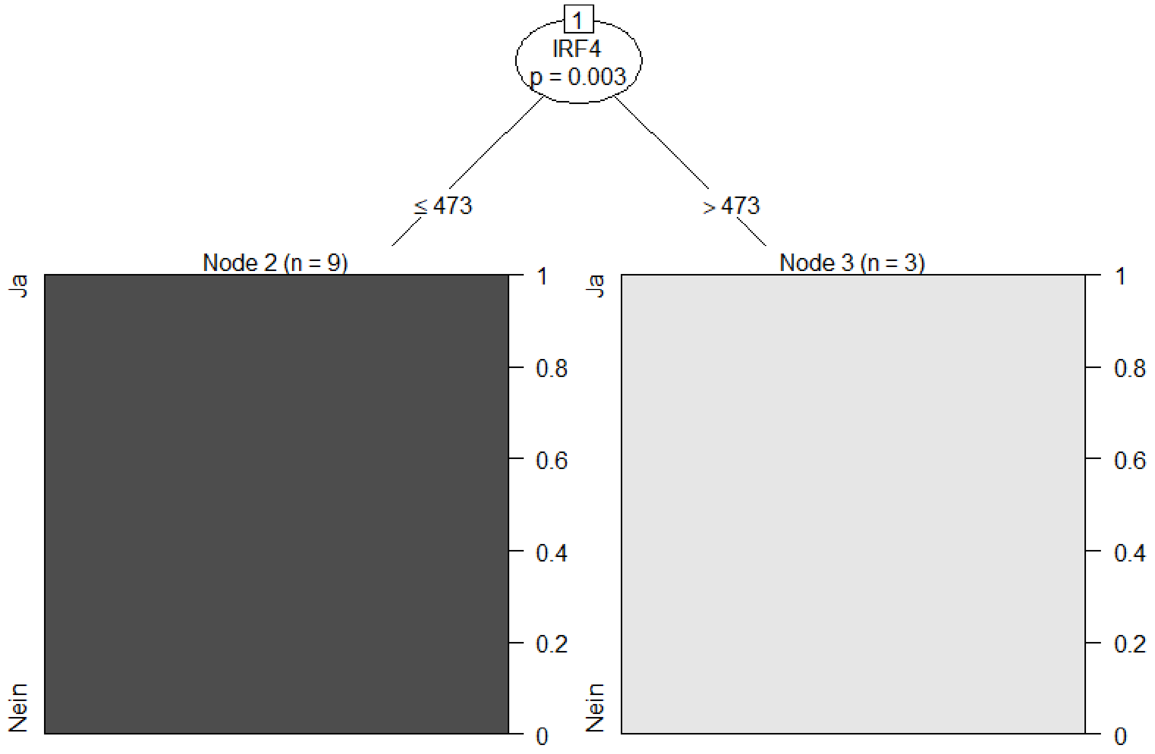

Figure S3. Also for IgG4-ROD, CIT revealed a one-tier system based on *IRF4* expression, with the appertaining cut-off of >473 counts identifying IgG4-ROD cases.
